# Food Plant Secondary Metabolites Antiviral Activity and Their Possible Roles in SARS-CoV-2 Treatment: An Overview

**DOI:** 10.3390/molecules28062470

**Published:** 2023-03-08

**Authors:** Deborah Giordano, Angelo Facchiano, Virginia Carbone

**Affiliations:** Institute of Food Sciences, National Research Council, via Roma 64, 83100 Avellino, Italy

**Keywords:** phytochemicals, secondary metabolites, antiviral activity, HIV, SARS-CoV-2, COVID-19, molecular simulations, in vitro assays, in vivo studies

## Abstract

Natural products and plant extracts exhibit many biological activities, including that related to the defense mechanisms against parasites. Many studies have investigated the biological functions of secondary metabolites and reported evidence of antiviral activities. The pandemic emergencies have further increased the interest in finding antiviral agents, and efforts are oriented to investigate possible activities of secondary plant metabolites against human viruses and their potential application in treating or preventing SARS-CoV-2 infection. In this review, we performed a comprehensive analysis of studies through in silico and in vitro investigations, also including in vivo applications and clinical trials, to evaluate the state of knowledge on the antiviral activities of secondary metabolites against human viruses and their potential application in treating or preventing SARS-CoV-2 infection, with a particular focus on natural compounds present in food plants. Although some of the food plant secondary metabolites seem to be useful in the prevention and as a possible therapeutic management against SARS-CoV-2, up to now, no molecules can be used as a potential treatment for COVID-19; however, more research is needed.

## 1. Introduction

**Figure 1:**
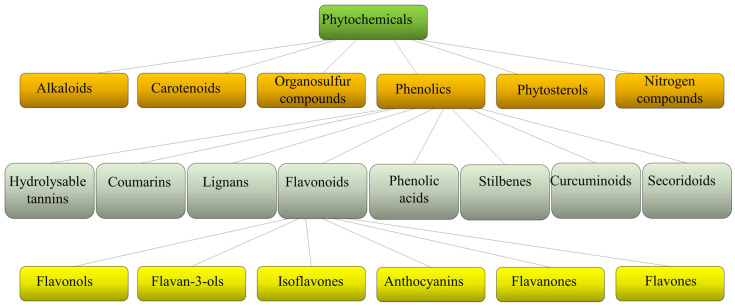
Schematic classification of phytochemicals, with sub-classification of phenolics and flavonoids.

Plants produce a variety of structurally diverse and often complex secondary metabolites (SM) with a range of biological functions [1]. Plant SM can be classified according to several criteria, such as chemical structure (presence of rings or sugars), composition (containing nitrogen or not), solubility in organic solvents or water, and the biosynthetic pathway [2]. They include alkaloids, carotenoids, organosulfur compounds, phytosterols, nitrogen compounds, and phenolics (Figure 1).

Each class is then divided into further classes. In particular, phenolics comprise a large group of different compounds, with the phenolic hydroxyl groups being the common structural feature. These are usually found conjugated with sugars and organic acids and can be divided into seven main classes such as hydrolyzable tannins, coumarins, lignans, phenolic acids, stilbenes, curcuminoids, and flavonoids [3] (Figure 1). Flavonoids exist broadly in nature, and to date, more than 9000 flavonoids have been reported. They can be divided into the following subclasses: flavonols, flavan-3-ols, isoflavones, anthocyanins, flavanones, and flavones. Flavonoids share a common structure of two benzene rings connected by three carbon atoms forming an oxygenated heterocycle and are responsible for the red, blue, and yellow coloration of plants and are found in foods and beverages of plant origin, such as fruits, vegetables, tea, cocoa, and wine [4,5]. Recent studies have reported that some of these natural compounds can reduce the risk of many chronic diseases and can significantly modulate and diversify the composition of the human gut microbiome [6]. Consumption of phenolic compounds can help to prevent metabolic, cardiovascular, respiratory, neurological, and cancerous diseases [7,8] due to their high antimicrobial, antioxidant, and anti-inflammatory immunomodulatory activities [9,10,11]. Eating plant-based foods is part of many diverse dietary patterns, including the well-studied Mediterranean diet [12], vegan, and vegetarian approaches. Despite the widely known health benefits of consuming fruits and vegetables, the intake is often inadequate and a large number of adults worldwide do not consume the daily-recommended servings. So, in the attempt to improve health, dietary guidelines and health promotion campaigns have advocated for individuals to “eat a rainbow” of fruit and vegetables, i.e., to take a qualitative color rather than a quantitative servings approach, based on the association of each color with a health benefit. For example, red foods include antioxidants that can contribute to decreased inflammation in the body; orange foods are abundant in carotenoids and have been linked to endocrine-regulating activities; yellow foods have been found to aid in digestion due to their fiber content and bioflavonoids that promote healthy gut bacteria; green foods, especially green leafy vegetables, contain an abundance of polyphenols that aid in reducing cardiovascular risk factors such as high blood pressure; blue or purple foods have been found to improve memory and mood because of flavonoids, flavonols, and phenolic acids that promote cognitive functions.

Recent technological advances in analytical methods such as metabolomics, metabolic engineering, and synthetic biology, as well as the ad hoc designed computational tools and databases, are providing powerful tools for drug discovery based on natural compounds [6]. It has been known that natural products and plant extracts exhibit potent anti-viral activities, often against multiple virus families, thus suggesting that they may be useful as broad-spectrum antiviral agents [13,14].

The COVID-19 pandemic caused by SARS-CoV-2 has prompted researchers to conduct many studies aimed at identifying useful compounds to counter the viral agent. As reported by the World Health Organization’s weekly epidemiological update dated 22 February 2023, “over 757 million confirmed cases and over 6.8 million deaths have been reported globally” [15], with nearly 5.3 million new cases and over 48,000 deaths in the 4 weeks preceding the report. For a state-of-the-art review of the pandemic two years after its appearance and the related lessons learned, an interesting article was published in September 2022 [16].

**Figure 2:**
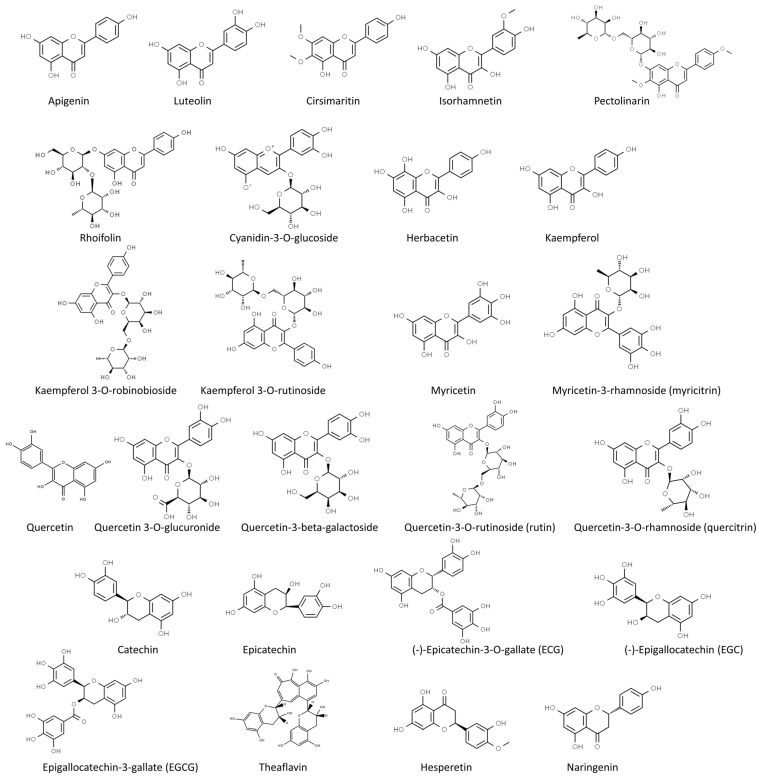
Chemical structure of the selected flavonoids (from ChemSpider, http://www.chemspider.com/ accessed on 24 February 2023).

**Figure 3:**
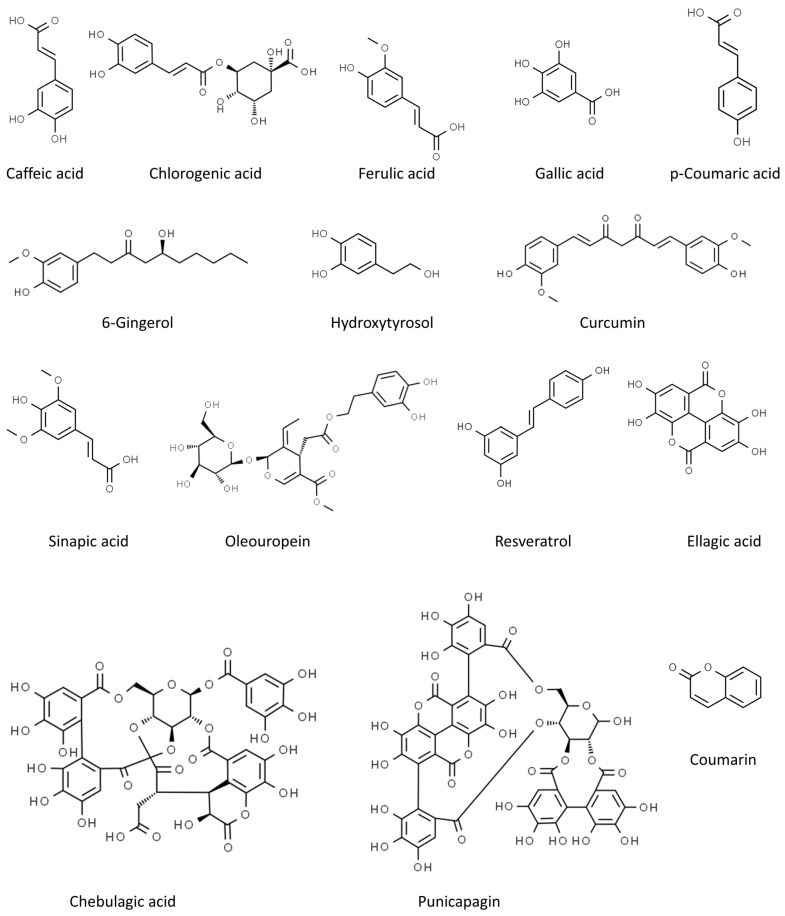
Chemical structure of the other selected phenolics (from ChemSpider, http://www.chemspider.com/ accessed on 24 February 2023).

**Figure 4:**
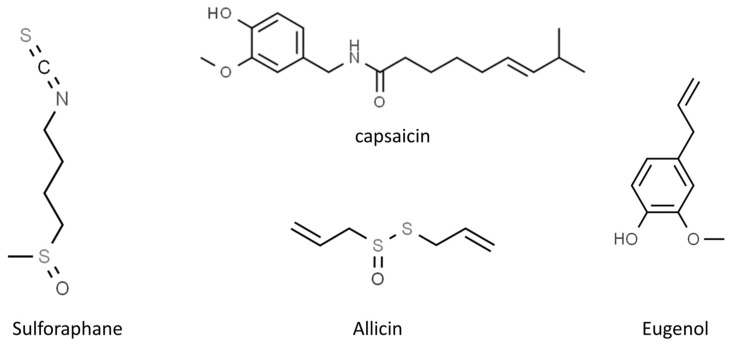
Chemical structure of the other selected phytochemicals (from ChemSpider, http://www.chemspider.com/ accessed on 24 February 2023).

In our review, we summarize the progress of studies on the antiviral activities of secondary metabolites and, in particular, we focus on 45 widely studied natural compounds present in food plants selected from the literature on the basis of their activity against human viruses and their potential application in treating or preventing SARS-CoV-2 infection, as demonstrated through in silico and in vitro studies, including also any in vivo application. The list of 45 phytochemicals and their related food source is shown in Table 1. Their chemical structures are shown in Figure 2, Figure 3 and Figure 4.

## 2. Antiviral Potential of Selected Natural Phytochemicals

Natural products serve as excellent sources for discovering antiviral agents due to their diversity and complexity and can offer remarkable efficacy and specificity to target viral infections [118]. The antiviral activity of a drug can affect viral entry, viral DNA and RNA synthesis, or viral reproduction, and is conditioned by viral structure and its replication cycle. For example, the presence or absence of a viral envelope, and, consequently, the different modes of entry of the virus into the host cell, play a significant role in the effectiveness of the virucidal activity, as the surface of the external membrane of the virus establishes the first contact with the drug. The antiviral activity of secondary metabolites can be evaluated by various biological assays to test the cytotoxicity, cytopathic effect inhibition, and the capability to block viral spread from cell to cell, thus limiting and/or fighting viral circulation [132]. Several natural products exhibit antiviral activities towards DNA and RNA viruses by acting on different cellular/viral targets and interfering with the infection and the viral replication cycle. Many of the secondary metabolites that are object of this review have shown antiviral activity against the *Herpes simplex virus* (HSV). HSV belongs to Herpesviridae, which is a broad family of enveloped-DNA viruses and is categorized into two types: HSV-1, commonly associated with orofacial ulcers, and HSV-2, which mainly causes genital ulcers [101]. For example, caffeic acid inhibits *Herpes simplex type 1* (HSV-1) multiplication mainly before the completion of viral DNA replication but not thereafter [17]. Gallic acid, also belonging to the class of phenolic acids, has shown activity against HSV [29]. In the context of flavonoids, luteolin—belonging to the class of flavones—exhibited potent antiviral activity against wild-type and clinical isolates of HSV-2 [43], as well as kaempferol and its glycosides kaempferol-3-*O*-robinobioside and kaempferol-3-*O*-rutinoside, myricetin, quercetin, and its glycosides quercetin-3-*O*-rutinoside (rutin), and isorhamnetin, all belonging to the class of flavonols that have shown inhibitory activity against HSV-1 and/or HSV-2 [29,38,39,60,71]. The flavanols epicatechin, (-)-epicatechin-3*-O-*gallate (ECG), (-)-epigallocatechin (EGC), and epigallocatechin-3-gallate (EGCG) and the flavanone naringenin possess antiherpetic activity [29,83]. In vitro studies have also been performed on molecules belonging to other classes of phenols such as curcumin [101], resveratrol [105], chebulagic acid [113], and other secondary metabolites such as allicin [126] and eugenol [128], demonstrating their antiviral activity against HSV-1 and HSV-2.

Furthermore, studies have investigated the antiviral activity of some secondary metabolites against the Hepatitis C virus (HCV), a virus belonging to the Flaviviridae family that represents one of the major causes of chronic liver disease. It has been demonstrated that caffeic acid, as well as the flavone apigenin, could inhibit HCV replication [19,38], while gallic acid, quercetin-3-*O*-rutinoside (rutin), epigallocatechin-3-gallate (EGCG), and curcumin could inhibit HCV entry [25,77,83,101]. In hepatitis virus infection, coumarin has been shown to target a wide range of proteins, like binding antigens present at the cell surface, proteins involved in viral replication, and interferon signaling pathways [44]. Furthermore, it has been demonstrated that sulforaphane, an isothiocyanate widely distributed in Brassicaceae plants, suppresses the replication of HCV by inducing the heme-oxygenase-1 (HO-1) expression, an enzyme that interferes with the replication of the virus through the activation of nuclear factor (erythroid-derived 2)-like (Nrf2) pathway, which regulates the expression of antioxidant proteins [124].

Adenoviruses (ADVs) are DNA viruses that typically cause mild infections involving the upper or lower respiratory tract, gastrointestinal tract, or conjunctiva. More than 50 serotypes have been identified showing different tissue tropisms that correlate with clinical manifestations of infection [133]. In a study conducted to examine the antiviral activity of aqueous extract and pure compounds of *Plantago major* L., a popular traditional Chinese medicine, it was found that chlorogenic acid was active against ADV-3, ADV-8, and ADV-11; instead, caffeic acid was active against ADV-3 [18]. Epigallocatechin-3-gallate (EGCG) has been shown to reduce the virus titers of adenovirus in two cell infection models, to inactivate purified adenovirions and to inhibit the attachment of adenovirus by interacting with virion surface proteins [83]. Caffeic acid also inhibits the multiplication of the Influenza A (IAV) virus in the early stage of viral multiplication [20]. Influenza virus (IV) is a single-stranded RNA virus, which belongs to the Orthomyxoviridae family, that causes acute respiratory infection. There are four types of influenza viruses: A, B, C, and D. Human influenza A and B viruses cause seasonal epidemics of disease; influenza C virus infections generally cause mild illness and are not thought to cause human epidemics; influenza D viruses primarily affect cattle and are not known to infect or cause human illness. Type A virus is divided into subtypes according to the antigenic properties of surface glycoproteins, hemagglutinin (HA), and neuraminidase (NA). In relation to genetic and antigenic variation, the current 18 subtypes of IAV HA (H1–H18) are divided into group 1 (H1, H2, H5, H6, H8, H9, H11, H12, H13, H16, H17, and H18) and group 2 (H3, H4, H7, H10, H14, and H15), while IAV of NA has 11 subtypes (N1–N11) [134]. Gallic acid has been shown to exhibit inhibitory effects against both influenza type A and B viruses, disrupting the viral particles [28], while hydroxytyrosol inactivates influenza A viruses, including H1N1, H3N2, H5N1, and H9N2 subtypes with an effect virucidal rather than antiviral [35]. Apigenin, luteolin, kaempferol, epicatechin, (-)-epicatechin-3*-O-*gallate (ECG), epigallocatechin-3-gallate (EGCG), cirsimaritin, and eugenol also show activity against IV inhibiting replication [38,39,50,83,129]. An in vitro and in vivo study demonstrated that isorhamnetin exerts anti-influenza effects via direct HA and NA inhibition, direct or indirect inhibition of the expression of viral HA and NA genes as well as reduces virus-induced ROS (reactive oxygen species) generation [53]. Several in vitro studies demonstrated that curcumin inhibits the uptake, replication, and particle production of different IAVs [101]. In an in vitro and in silico study, Sang et al. [63] demonstrated that myricetin has not only a direct antiviral activity against IAV-inhibiting virus replication but also has immune modulatory effects. Wu et al. [73] demonstrated that quercetin inhibits a wide spectrum of influenza groups, including H1N1, H3N2, and H5N1, binding to influenza hemagglutinin protein and inhibiting viral-cell fusion.

Instead, quercetin 3-rhamnoside (quercitrin) has a strong antiviral activity against the influenza A/WS/33 (H1N1) virus by inhibiting replication in the initial phase of the infection by indirect interaction with viral particles [78]. Sahoo et al. [87] used in silico docking approaches to explore the molecular interaction of 13 active compounds extracted from plants against NA protein of H1N1 and observed that theaflavin, found in green tea, could be a potential inhibitor of H1N1 NA proteins, as strongly suggested by lowest docking energy. Molecular docking studies have also shown the inhibitory role against IV infection of coumarin and its derivatives that can target various enzymes and pathways essential for viral entry, survival, and infection [44]. An in vitro study on the effect of sulforaphane on IAV replication in Madin-Darby canine kidney cells showed a decrease in replication [124].

The Human immunodeficiency virus (HIV), a human retrovirus belonging to the lentivirus family that attacks the body’s immune system, is responsible for causing acquired immunodeficiency syndrome (AIDS) once the immune system is compromised. There are two known types of HIV, which are deeply related, named HIV-1 and HIV-2. HIV-1 is the main cause of AIDS and responsible for the worldwide HIV pandemic; HIV-2, which differs in genomic structure and antigenicity, also causes the disease but with slower progression [135]. Different plants and phytochemicals exert activity against HIV. For example, gallic acid and ellagic acid possess anti-HIV activity and may act as post-entry inhibitors by affecting HIV protease activity [26]. Hydroxytyrosol also shows anti-HIV activity in vitro, inhibiting the viral integrase enzyme and fusion of the viral envelope with host cells [34]. Apigenin, as well as epicatechin, (-)-epicatechin-3*-O-*gallate (ECG), and epigallocatechin-3-gallate (EGCG), exhibit antiviral activities against HIV, presumably inhibiting HIV-1 protease enzyme and HIV reverse transcriptase [38,83]. Luteolin inhibits HIV-1 at the transcription level after the viral integration step [39], while myricetin inhibits the HIV-1 integrase (IN) and the HIV-1 reverse transcriptase [62]. The results from Ortega and co-workers [68] showed that the differences in the antiviral activity and the RT inhibition of the myricetin and myricetin-3-rhamnoside (myricitrin) together with the in silico analysis suggested that the glycosyl moiety could play a role in the entry of flavonoids into the cell and then, after enzymatic cleavage of the glycosyl moiety, the myricetin aglycone would ultimately be responsible for the anti-HIV activity. Quercetin has been investigated in vitro as an antiviral agent for HIV due to its ability to inhibit crucial enzymes such as reverse transcriptase, integrase, and protease [72]. Resveratrol also has effects on viral infection by inhibiting HIV-1 strain replication [39]. Several studies have reported that curcumin exhibits anti-HIV activity by directly targeting viral proteins. In particular, curcumin shows inhibition of the HIV-1 and HIV-2 proteases, the HIV integrase and the HIV trans-activator of transcription (tat), a viral transcription regulator [101]. Coumarin derivatives also exhibit their anti-HIV activity by targeting viral proteins inhibiting HIV protease, integrase, reverse transcriptase, and tat but also by inhibiting viral DNA replication [44]. Sulforaphane protects macrophages from HIV infection by mobilizing the transcription factor and antioxidant response regulator nuclear factor E2-related factor 2 (Nrf2) [124].

Enterovirus 71 (EV71), which belongs to the genus *Enterovirus*, is the major pathogen of hand, foot, and mouth disease (HFMD) that often occurs in children under the age of 10 [27]. Gallic acid was found to exert strong anti-EV71 activity [27], while isorhamnetin inhibits EV71 RNA replication and protein synthesis [38]. It has been demonstrated that resveratrol inhibits EV71 replication [106]. Antiviral activity of epigallocatechin-3-gallate (EGCG) and coumarin and its derivatives has been observed against EV71 [44,83]. Chebulagic acid and Punicalagin exhibit antiviral activity both in vitro and in vivo against EV71 [112,115].

In addition to the previously described viruses, phytochemicals included in this review also inhibit a wide range of other viruses, as summarized in Table 1.

## 3. Coronaviruses and Phytochemicals

Coronaviruses (CoVs) were identified as human pathogens in the 1960s. They are enveloped, positive-stranded RNA viruses in the order of Nidovirales [136], which surface has a crown-like appearance that can be seen under the electron microscope (from the Latin word corona, meaning crown or halo). There are currently seven coronaviruses known to infect humans and four of them cause mild-to-moderate disease: HCoV-OC43, HCoV-HKU1, and HCoV-229E cause common colds (but may cause severe lower respiratory tract infections among people in the youngest and oldest age groups), while HCoV-NL63 is an important cause of (pseudo) croup and bronchiolitis in children [137]. The other three coronaviruses cause more severe and possibly even fatal diseases and have emerged more recently: SARS-CoV, responsible for the severe acute respiratory syndrome (SARS), which emerged in 2002; MERS-CoV, responsible for Middle East respiratory syndrome (MERS), which emerged in 2012; and SARS-CoV-2, responsible for coronavirus disease 2019 (COVID-19), which was identified in late 2019 [138,139].

The COVID-19 pandemic is an unprecedented global health crisis caused by a novel coronavirus, which was scientifically named SARS-CoV-2 of the Coronaviridae family. This virus was categorized as a member of *Betacoronavirus* genus alongside SARS-CoV and MERS-CoV [140].

**Figure 5:**
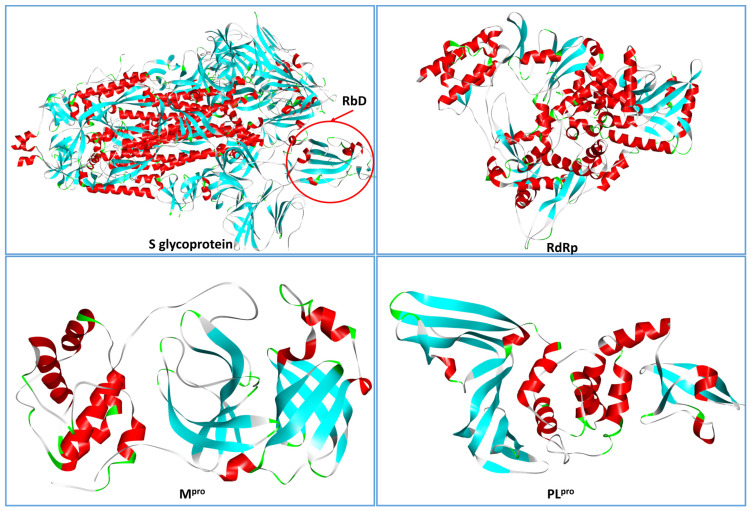
The four main proteins of SARS-CoV-2 represented by the backbone ribbon. Colors refer to helices (red), beta strands (cyan), turns (green), and generic loops (grey). Images are generated by DiscoveryStudio 4.5 (Dassault Systèmes, France).

SARS-CoV-2 contains a positive-sense single-stranded RNA genome of around 30 kb that consists of several open reading frames (ORFs). Two-thirds of viral RNA, mainly located in the first ORF (ORF1a/b), translates two polyproteins, pp1a and pp1ab, that are cleaved by the viral papain-like protease (PL^pro^) and the 3C-like cysteine protease (3CL^pro^), also known as main protease (M^pro^), to produce the non-structural proteins (NSP) 1–16, while the remaining ORFs encode accessory and structural proteins. The remaining part of the viral genome encodes four essential structural proteins, including spike (S) glycoprotein, small envelope (E) glycoprotein, membrane (M) glycoprotein, and nucleocapsid (N) protein, and also several accessory proteins that interfere with the host innate immune response [141]. Like all coronaviruses, SARS-CoV-2 utilizes the S glycoprotein to mediate entry into the host cell. This protein is composed of two subunits: the S1 subunit, which consists of the receptor-binding domain (RBD) and the N-terminal domain (NTD), and the S2 subunit, which mediates the fusion of the viral and host cell membranes [142]. SARS-CoV-2 virus enters the cells of the respiratory system by interacting with angiotensin-converting enzyme 2 (ACE2) receptors at the surface of host cells. The ACE2 receptor is highly expressed throughout the respiratory tract cells, such as nasal epithelial cells, goblet/secretory cells, and type II pneumocytes of the lung [117]. SARS-CoV-2 S protein binds to the ACE2 receptor at the surface of host cells, initially through the S1 RBD. S1 is then shed from the viral surface, allowing S2 to fuse to the host cell membrane. The process of membrane fusion depends on host cell furin proteases and transmembrane serine protease 2 (TMPRSS2) that are responsible for S glycoprotein cleavage at S1/S2 and S2′ sites. In particular, furin cleavage at the S1/S2 site may lead to conformational changes in the viral S protein that exposes the RBD and/or the S2 domain, while TMPRSS2 cleavage of the SARS-CoV-2 S protein is believed to enable the fusion of the viral capsid with the host cell to permit viral entry [143,144]. In order to replicate the RNA genome, the virus encodes an RNA-dependent RNA Polymerase (RdRp). RdRp is composed of three different nonstructural proteins: the catalytic subunit NSP12 and its two accessory subunits NSP7 and NSP8 [145]. Potential therapies for SARS-CoV-2 can be different based on the targets since some drugs can target the virus while others can target the host and its immune system. As S glycoprotein, M^pro^, PL^pro^, and RdRp (Figure 5) are essential for virus replication, they represent suitable therapeutic targets.

Some natural constituents found in food of plant origin and dietary supplements demonstrate activity against SARS-CoV-2 by affecting the production of cytokines, modulating cell signaling pathways related to inflammation, and even by direct interaction with targets found in the virus. This has been demonstrated by the application of in vitro techniques and in silico molecular docking studies that estimate binding strength.

There are some studies reporting the antiviral activity of phytochemicals against human coronaviruses. For example, Weng et al. [21] examined the potent anti-HCoV-NL63 activity of Sambucus FormosanaNakai extract, a traditional medicinal herb belonging to the Adoxaceae family, an elderberry species. Caffeic acid, chlorogenic acid, and gallic acid, which were reported as the phenolic acid constituents in this extract, were subjected to plaque formation, virucidal activity, and virus attachment assays in order to assess their antiviral mechanism against HCoV-NL63. All three phenolic acids exhibited the ability to reduce the progeny production of HCoV-NL63 particles in vitro, mainly caffeic acid, which resulted in a noteworthy reduction of virus yield (IC_50_ = 3.54 μM), plaque formation (5.4 μM), and virus attachment (IC_50_ = 8.1 μM). It has been demonstrated that apigenin inhibits SARS-CoV 3CL^pro^ while luteolin binds to the surface of the SARS-CoV spike protein and inhibits the virus entry into host cells [38].

In another study, SARS-CoV 3CL^pro^ inhibitory compounds were investigated by Jo et al. [47] employing the proteolytic technique against a flavonoid library by utilizing a synthetic peptide labeled with an Edans-Dabcyl FRET (Fluorescence resonance energy transfer) pair. In the flavonoid’s library, three flavonoids, i.e., herbacetin, rhoifolin, and pectolinanrin, were effective at inhibiting SARS-CoV 3CL^pro^ enzyme activity by reducing the fluorescence intensity. The IC_50_ value of herbacetin, rhoifolin, and pectolinanrin was calculated by the dose-dependent inhibitory curve and was found to be 33.17 µM, 27.45 µM, and 37.78 µM, respectively. Induced-fit docking experiments indicated that the S1 and S2 sites are involved in the binding of herbacetin due to the extra 8-OH group, which was predicted to develop increased binding affinity at these sites. On the other hand, rhoifolin and pectolinarin also showed higher binding affinity around the S1 and S2 sites, but the carbohydrate groups of these glycosylated flavonoids were responsible for this affinity. Rhoifolin’s higher binding affinity might be due to the coordinated binding across the S1, S2, and S3 sites. Yu et al. [64] evaluated the potential inhibitory effects of sixty-four purified bioactive compounds against the activity of SARS-CoV helicase NSP13 by an in vitro approach and found that myricetin inhibited the SARS-CoV helicase protein by affecting ATPase activity. In another study, quercetin-3-β-galactoside, a quercetin derivative, was identified as an inhibitor of SARS-CoV 3CL^pro^ using mutagenesis studies, molecular docking, surface plasmon resonance, and fluorescence resonance energy transfer bioassays showing an inhibitory activity with an IC_50_ of 42.79 µM. The molecular-modeling-driven biosynthesis of eight new quercetin-3-β-galactoside derivatives and subsequent 3CL^pro^ inhibition studies revealed the importance of hydroxyl groups of the quercetin moiety in 3CL^pro^ inhibition since their removal caused a substantial reduction in the inhibitory activity [76]. As evidenced in a study, *Isatis indigotica* root extract, as well as plant-derived phenolic compounds, were able to inhibit the cleavage of SARS-CoV 3CL^pro^ in a cell-free assay. Among them, hesperetin presented an IC_50_ value of 18.1 µg/mL and showed inhibition with IC_50_ value of 2.5 µg/mL when tested in a cell-based assay [92].

## 4. Natural Phytocompounds with Potential to Inhibit the Coronavirus SARS-CoV-2 According to In Silico Approaches

In silico molecular modeling uses the crystal structure of viral targets from SARS-CoV-2, or targets of related viruses such as SARS-CoV, docked with molecules under study to establish the binding strength of competitive inhibitors of the target that is expressed in terms of Kcal/mol. The binding strength of a test compound is compared with that of a known inhibitor or control. The in silico docking is no indication that a compound will absolutely inhibit a target, but it offers a good start for engaging in drug design [146]. In this review, we have performed an analysis of studies carried out with an in silico approach of possible interactions of the same molecules analyzed above for their antiviral activity with the major targets of SARS-CoV-2, like the main protease (M^Pro^), RdRp, membrane (M) glycoprotein, envelope (E) glycoprotein, spike (S) glycoprotein, PL^pro^, non-structural proteins (NSP) and ACE2 receptor. In particular, for those molecules that have shown activity against SARS-CoV, it has been postulated that they could potentially act against SARS-CoV-2, taking into account that SARS-CoV and SARS-CoV-2 have a high sequence similarity (79.5%) [147]. For example, Alrasheid and co-workers [30] have conducted an in silico study by using Molecular Operating Environment (MOE) drug discovery software platform in order to evaluate the antiviral activity targeting SARS-CoV-2 of twenty-one compounds from medicinal plants. They have found that gallic acid, quercetin, and capsaicin were among the best compounds interacting with SARS-CoV-2 M^pro^, with rank scores ranging from −17.45 to −13.90 Kcal/mol. Moreover, a screening via molecular docking to test the binding affinity of various selected bioactive compounds of honey and propolis as inhibitors against the SARS-CoV-2 M^pro^ and RdRp revealed that ellagic acid and kaempferol have the strongest interaction with RdRp, instead the previously cited compounds and quercetin with the SARS-CoV-2 M^pro^ [32]. In another study, in which one hundred secondary metabolites from *Aframomum melegueta*, a Zingiberaceae family plant spice widely spread in Africa, have been computationally evaluated for inhibition of SARS-CoV-2 targets, it is reported that quercetin could be a potential SARS-CoV-2 2′-O-methyltransferase (NSP16) enzyme inhibitor while apigenin could inhibit SARS-CoV-2 main protease (M^pro^) [41]. In a 2020 study published as a preprint, kaempferol, quercetin, naringenin, oleuropein, curcumin, catechin, and epicatechin-gallate appeared to have the best potential to act as SARS-CoV-2 M^pro^ inhibitors with binding energies values obtained from the docking of main protease of −8.58, −8.47, −7.89, −7.31, −7.05, −7.24, and −6.67 Kcal/mol, respectively [57]. Although the preprint was not published elsewhere to date, other studies confirm the results. Bilginer et al. [58] also showed similar data for kaempferol, quercetin, and oleuropein; instead, Halder et al. [81] for curcumin and catechin. Moreover, Mukheriee et al. [59] confirmed a possible binding of epicatechin-gallate, quercetin, and kaempferol to the M^pro^ active site, with binding energies values of −8.5, −7.6, and −7.3 Kcal/mol respectively, and underline as epigallocatechin-gallate shows the best affinity in comparison to the re-docked inhibitor binding energy (−7.7 Kcal/mol).

In another in silico study, published as a preprint, luteolin was also screened against SARS-CoV-2 main protease (M^pro^), showing binding energy values of −8.35 Kcal/mol [44]; also, in this case, further studies corroborate this hypothesis [45]. Based on the inhibitory activity against SARS-CoV 3CL^pro^ exerted by pectolinarin, rhoifolin, and herbacetin [47,49] performed an in silico docking study to deduce their binding mode and binding affinity with SARS-CoV-2 3CL^pro^ and found that the affinity of rhoifolin became weakened while the efficiency of herbacetin and pectolinarin was still promising. Vicidomini et al. [54] found that for several flavonols and flavonol glucosides isolated from *Opuntia ficus-indica*, a dicotyledonous angiosperm cactaceous plant widespread worldwide in tropical and subtropical regions, including isorhamnetin, possible affinities for SARS-CoV-2 M^pro^, with binding energies lower than −7.0 Kcal/mol. Myricetin-3-rhamnoside (myricitrin) was found to have a strong binding with the active site residues of the SARS-CoV-2 M^pro^ with a binding score of −8.9 Kcal/mol. In particular, its active site residues Tyr54, Phe140, Gly143, His163, and Glu166 are involved in the formation of 6 hydrogen bonds with hydroxyl and carboxyl oxygen of ligand while His41 and Cys145 form Pi-alkyl and Pi-sulfur interactions with the aromatic scaffold of ligand, respectively. Other additional interactions, such as van der Waals forces, stabilize the binding of the ligand to the enzyme [69]. In a study in which thirty-eight flavonoids have been tested by molecular docking against the active site of the SARS-CoV-2 M^pro^, it has been demonstrated that natural aglycone flavonoids possess higher docking energies than flavonoids with sugar moieties. As a matter of fact, in these docking experiments, the aglycone myricetin displays a binding score of −7.4 Kcal/mol while myricetin-3-rhamnoside (myricitrin), also in this study, displays a binding score of −8.9 Kcal/mol. Similarly, quercetin-3*-O-*rhamnoside (quercitrin), quercetin-3-*O*-rutinoside (Rutin), quercetin-3-beta-galactoside, and quercetin 3*-O-*β-glucuronide exhibit lower binding energies than their aglycone quercetin (binding score of −9.7, −9.2, −8.4, −8.1, −7.5 Kcal/mol, respectively) [65]. Catechins and some of their derivatives showed a strong affinity for SARS-CoV-2 M^pro^ because they could form a stable ligand-receptor complex, and this leads to the hypothesis of a potential antiviral activity [79]. An in silico analysis has depicted that ellagic acid, epicatechin, and capsaicin exhibit significant binding and interaction with the most vital active site residue Cys145 of SARS-CoV-2 M^pro^ and can inhibit its activity in an extremely effective manner [82]. Ghosh et al. [80] selected eight polyphenols from green tea and elucidated the binding affinities and binding modes between these polyphenols and SARS-CoV-2 M^pro^ using molecular docking studies. All eight polyphenols exhibit good binding affinity, including catechin and (-)-epigallocatechin (EGC), but epigallocatechin-3-gallate (EGCG) and (-)-epicatechin-3*-O-*gallate (ECG) interact strongly with one or both the catalytic residues His41 and Cys145 of SARS-CoV-2 M^pro^ with a binding score of −7.6 and −8.2 Kcal/mol. Another study aimed to evaluate bioactive compounds found in plants to interact and hopefully inhibit SARS-CoV-2 M^pro^ and spike (S) glycoprotein using a molecular docking approach reported that pectolinarin, epigallocatechin-3-gallate (EGCG) and rhoifolin are among potential candidates to bind spike (S) glycoprotein surface, while rhoifolin and pectolinarin are among the candidates to become drugs targeting SARS-CoV-2 M^pro^ [48]. The role of tea polyphenols in prophylaxis and treatment of COVID-19 has been investigated by Mhatre et al. [84] in a preliminary in silico study in which molecular docking interactions of two tea polyphenols with some of the possible binding sites of SARS-CoV-2 were performed. In particular, the receptors 3CL^pro^, RdRp, PL^pro^, spike (S) glycoprotein RBD, and ACE2 receptor with spike (S) glycoprotein RBD were docked against epigallocatechin-3-gallate (EGCG) from green tea. For all the receptors studied, EGCG exhibited good docking scores with various types of interactions, and except for RdRp, the binding energies were consistently better than −8.0 Kcal/mol. Mostafa et al. [88] have conducted an in silico molecular docking experiment on the effects of some natural antiviral phytoconstituents on the crystal structure of SARS-CoV-2 M^pro^. Many of the docked compounds revealed good binding affinity, with theaflavin showing a good binding score (−8.5 Kcal/mol). The bioactive phenolic phytocompound 6-gingerol, found in the fresh ginger rhizome, was also tested against different viral proteins by using the molecular docking technique in order to evaluate its interaction with SARS-CoV-2 targets. This study suggests a high binding affinity and interaction with surface regions of multiple targets of COVID-19, including viral proteases, RdRp, and spike (S) glycoprotein [99]. Du et al. [114] investigated in vitro and in silico the potent antiviral activity of chebulagic acid and punicalagin against SARS-CoV-2 viral replication. In silico docking of chebulagic acid and punicalagin to SARS-CoV-2 M^pro^ was performed in order to search potential allosteric binding sites, and both chebulagic acid and punicalagin may interact with the cleft between domain II and domain III within SARS-CoV-2 M^pro^ with stable binding free energy.

It is reported that SARS-CoV-2 can bind to Toll-like receptor 4 (TLR-4), which would eventually lead to α-synuclein aggregation in neurons and stimulation of neurodegeneration pathways. Oleuropein was investigated against the SARS-CoV-2 target (main protease 3CL^pro^), TLR-4, and Prolyl Oligopeptidases (POP), to explore oleuropein potency against the neurological complications associated with COVID-19. POP inhibition could reduce aggregation of alpha-synuclein in neurons, hence, investigating anti-COVID agent(s) against TLR-4 and POP might help in alleviating neurological complications linked with COVID-19. Docking analyses showed a binding score of −7.8, −8.3, and −8.5 Kcal/mol for oleuropein-3CL^pro^, oleuropein-TLR4, and oleuropein-POP interactions, respectively, suggesting oleuropein as a potential candidate that can target SARS-CoV-2 and alleviate neurological manifestations associated with it [119]. In a recent study, the antiviral potential of 50 natural coumarin phytochemicals isolated from plants was investigated in order to identify the binding interactions of these phytochemicals against the coronavirus 3CL^pro^ by molecular modeling approaches. It was found that glycycoumarin, Inophyllum P, mesuol, and oxypeucedanin hydrate displayed the highest binding affinity with the best negative energy scores and interacted with one or both of the catalytic residues (His41 and Cys145) of 3CL^pro^ through hydrophilic and hydrophobic bonding [121]. Shekh et al. [127], using virtual screening methods, have analyzed allicin to assess its ability to covalently modify cysteine residues of SARS-CoV-2 M^pro^. The results suggest that allicin may induce dual S-thioallylation of Cys145 and Cys85/Cys156 residues of SARS-CoV-2 M^pro^ and may be useful in attenuating the coronavirus infection. It is known that essential oils have anti-inflammatory, immunomodulatory, bronchodilatory, and antiviral properties and, for this reason, are being proposed to have activity against SARS-CoV-2 virus. Moreover, owing to their lipophilic nature, essential oils are advocated to penetrate viral membranes easily, leading to membrane disruption. Molecular docking techniques were applied to screen the anti-SARS-CoV-2 efficacies of eugenol, menthol, and carvacrol, major components of essential oils, against various protein targets of SARS-CoV-2. Results revealed that these compounds have binding affinities towards SARS-CoV-2 spike (S) glycoprotein, SARS-CoV-2 M^pro^, RdRp, and human ACE2 proteins, respectively [128].

Paolacci and co-workers [36] investigated the potential effects of hydroxytyrosol in combination with alpha-cyclodextrin, another naturally occurring compound, on SARS-CoV-2 entry into human cells. Bioinformatic docking studies showed that hydroxytyrosol is captured in the hydrophobic cavity of alpha-cyclodextrin, and the resulting complex could interact with the SARS-CoV-2 spike (S) glycoprotein and its host cell receptor ACE2. Even if these bindings do not occur at the spike-ACE2 interface, they may induce structural perturbations that could potentially influence the endocytosis process. Sekiou et al. [51] have screened in silico the interaction between the main protease SARS-CoV-2 M^pro^ active site with natural compounds, including cirsimaritin and quercetin, and found that both have a better binding affinity to this target. Molecular docking was used by Pandey et al. [39] to describe the binding ability of naturally occurring phytochemicals, including kaempferol and quercetin, with SARS-CoV-2 spike (S) glycoprotein and found that these natural compounds are capable of binding to either the S1 or S2 domains of the SARS-CoV-2 S, most probably preventing it from binding to the ACE2 receptor or internalization during fusion.

High-end molecular docking analysis and MD simulation were performed to characterize the binding affinity of natural and synthetic anti-viral compounds with the SARS-CoV-2 structural proteins and to analyze the stability of drug-protein interactions. Results identified rutin as a potent inhibitor of SARS-CoV-2 envelope (E) glycoprotein, while caffeic acid and ferulic acid were found to inhibit SARS-CoV-2 membrane (M) glycoprotein [22]. Orfali et al. [33] screened all available SARS-CoV-2 molecular targets using a multistep in silico protocol to find out the most probable one that mediates the proved in vitro anti-SARS-CoV-2 activity of sinapic acid and found that the viral envelope protein (E-protein) was suggested as the most probable hit for sinapic acid. A further in-depth molecular dynamic simulation-based investigation was performed to precisely explore the binding path and mode of sinapic acid with E-protein.

The interactions of the two crucial proteins NSP9 and NSP10 of COVID-19 have been investigated with potential antiviral compounds from *Moringa oleifera* using molecular docking and dynamic methods. The results revealed that all selected ligands form stable complexes with the targeted proteins and showed the highest binding affinity values of apigenin (−7.1 Kcal/mol) for NSP10 and ellagic acid (−7.1 Kcal/mol) for NSP9 [40]. Several viral proteins have been found to possess deubiquitinating activity, and they can be used to antagonize or modulate the antiviral immune signaling pathway. Along with protease activity, SARS-CoV-2 PL^pro^ possesses deubiquitinating activity. Naphthalene-based inhibitors, such as the well-investigated GRL-0617 compound, have been shown to possess dual effects, inhibiting both protease and deubiquitinating activity of the PL^pro^. Pitsillou et al. [52] investigated the binding characteristics of the same dietary compounds, including rutin and cyanidin-3*-O-*glucoside, to the PL^pro^ and evaluated the deubiquitinating activity. Analyses of molecular docking highlighted the relatively high affinity of GRL-0617 and dietary compounds. A study was conducted on the most abundant pomegranate (*Punica granatum*) peel extract constituents, including punicalagin, with the aim of exploring their anti SARS-CoV-2 properties using in silico tools. The protein targets used in this study were: SARS-CoV-2 spike (S) glycoprotein, ACE2, furin, and TMPRSS2. Molecular docking results showed that all the analyzed ligands interacted through hydrogen bonds with amino acid residues at the S glycoprotein predicted druggable site, and punicalagin presented free binding energy of −7.312 Kcal/mol, with the ligand-protein complex stabilized through three hydrogen bonds (Asn343, Asn370, and Ser371). All tested pomegranate peel extract constituents showed significant binding affinity at the ACE2 predicted druggable site, with all ligand–protein complexes stabilized through hydrogen bonds, and punicalagin presented free binding energy of −7.144 Kcal/mol. Amino acid residue Lys441 was found to be important for the stabilization of punicalagin–ACE2 complex. Moreover, all pomegranate peel extract constituents formed stable complexes with furin, with punicalagin-free binding energy of −9.385 Kcal/mol. Punicalagin showed intensive interactions with TMPRSS2 amino acid residues at the predicted binding site with binding energy values of −7.358 Kcal/mol and with four hydrogen bonds. Polar interactions with Asn97 and Arg405 residues were essential for the stabilization of TMPRSS2 complexes with punicalagin [117]. Özdemir et al. [122] carried out molecular docking studies on five different proteins (Spike S1-subunit, NSP5, NSP12, NSP15, and NSP16) of the SARS-CoV-2 and two proteins (ACE2 and Vitamin K epoxide reductase complex subunit 1-VKORC1, a key enzyme in recycling reduced vitamin K, that plays an essential role in γ-carboxylation of vitamin K-dependent coagulation factors) of human and found that the best binding scores for 17 coumarins were determined for NSP12, with the highest score (−10.01 Kcal/mol) showed by 2-morpholinoethan-1-amine substituted coumarin.

In conclusion, it is necessary to emphasize that the potential strength of molecular simulations offers the possibility of carrying out both large-scale screening and targeted docking simulations quickly and with limited costs, but on the other hand, it is also necessary to apply rigorous procedures and carefully evaluate the results obtained. The COVID-19 pandemic has prompted many research groups to carry out docking simulations to provide useful information to the scientific community as soon as possible and to identify possible solutions to the emergency as quickly as possible. However, we found several studies published only in pre-print form, and therefore without having received an evaluation by experts; others were published in peer-reviewed journals but very few days after submission to the journal. Although peer-reviewed, the very short evaluation time makes the quality of the review at least questionable. The result is that in several literature studies, as either full articles or pre-prints, there are poor protocols of docking simulations, weak or erroneous interpretations of the results, use of protein structures that are not experimental results but obtained by insufficiently described modeling procedures, docking with portions of proteins without critical evaluation of the real utility. In this review, we chose to quote only in silico simulation studies that meet sufficient quality criteria in the simulations performed and in the presentation and discussion of the results obtained.

## 5. In Vitro Evaluation of SARS-CoV-2 Antiviral Activity of Natural Phytocompounds

We carried out a screening of the literature in order to assess whether the SARS-CoV-2 antiviral activity postulated by in silico approaches for the natural compounds object of this review was also confirmed in studies carried out using in vitro approaches.

El Gizawy and co-workers [24] evaluated the in vitro anti-SARS-CoV-2 activities of four bioactive compounds isolated from the leaves of *Pimenta dioica* (L.) Merr (ferulic acid, rutin, gallic acid, and chlorogenic acid) that have already shown SARS-CoV-2 M^pro^ inhibitory activities using molecular docking and dynamics simulations. In order to confirm their findings, the compounds were tested for their half-maximal cytotoxicity (CC_50_) and SARS-CoV-2 inhibitory concentrations (IC_50_). Moreover, their anti-inflammatory effects on different cytokines and genetic markers were examined. Rutin, gallic acid, and chlorogenic acid have shown remarkable antiviral activities against SARS-CoV-2 at IC_50_ values of 31, 108, and 360 µg/mL (and CC_50_ values of 8017, 3108, and 3030 µg/mL), respectively, while the anti-inflammatory effects were found to be better in ferulic acid and rutin treatments. It is known that 10–20% of the individuals who have recovered from the SARS-CoV-2 infection are affected by post-acute COVID-19 syndrome, also known as long-COVID, a condition characterized by a complex of symptoms, and it has been hypothesized that multi-organ effects of long-COVID could be associated with the persistence of virus RNA/proteins in host cells. Crudele et al. [37] evaluated the pro-inflammatory effects of the expression of SARS-CoV-2 PL^pro^/NSP3 protein in a polarized human airway (Calu-3), intestinal (Caco-2) and liver epithelial (HepG2) cells, and next, whether the treatment with hydroxytyrosol is able to neutralize or reduce these effects. They demonstrated that SARS-CoV-2 PL^pro^ was able to induce a cascade of inflammatory genes and proteins and to increase the apoptotic rate and expression of several oxidative stress markers in all evaluated epithelial cells and that treatment with 10 µM hydroxytyrosol reverted SARS-CoV-2 PL^pro^-dependent effects almost completely. An in vitro study using fluorescence resonance energy transfer (FRET) assay was carried out on apigenin and quercetin in order to evaluate their ability to inhibit SARS-CoV-2 PL^pro^. FRET assay can be used to detect protein–protein interactions, and the results demonstrate that apigenin and quercetin are able to inhibit the 3CL^pro^ activity up to 92% and 52%, respectively, at 250 μg/mL. In order tofind the insight mechanism on how they inhibit the SARS-CoV-2 3CL^pro^ activity, in silico studies using molecular docking and structure-based pharmacophore were performed [42]. Luteolin and quercetin were tested in vitro against SARS-CoV-2 RdRp, and the IC_50_ value in the biochemical enzymatic assay is 4.6 ± 0.3 μM for luteolin and 6.9 ± 1.0 μM for quercetin. Potential binding modes of these compounds to the target protein were also investigated and these experimental and computational results complete previous computational investigations that proposed luteolin and quercetin as potential inhibitors of the crucial viral enzyme RdRp [46]. Jo et al. [49] conducted an in vitro analysis employing a proteolytic method to probe inhibitory compounds against SARS-CoV-2 3CL^pro^ by using a FRET assay. An in-house flavonoid library containing 5 isoflavones, 1 isoflavane, 18 flavones, 16 flavonols, 7 flavanols, 7 flavanones, 4 flavanonol, 1 prenylflavonoid, 9 chalcones, and 2 unclassified flavonoids were screened and, among them, rhoifolin, pectolinarin, herbacetin, and rutin were found to have inhibitory activity, with herbacetin and pectolinarin showing a prominent inhibitory activity (IC_50_ values of 53.90 and 51.64 µg/mL, respectively). The fluorescence spectra from tryptophans of SARS-CoV-2 3CLpro with candidate inhibitors were investigated to confirm these findings. As a matter of fact, tryptophan emits its fluorescence and, if it is properly located in the protein, the change in fluorescence intensity can reflect the binding state of the chemicals and can be used to determine the interaction between the protein and the chemical. Therefore, the decreased emission intensity confirmed the complex formation between the SARS-CoV-2 3CL^pro^ and the inhibitory compound. Pitsillou et al. [52] validated the results obtained by in silico studies on the inhibition of SARS-CoV-2 PL^Pro^ deubiquitinase activity of cyanidin-3*-O-*glucoside and rutin, using in vitro assays and the results showed that these compounds inhibit deubiquitinase activity in the micromolar range with rutin having a greater efficiency compared to cyanidin-3*-O-*glucoside. Using a SARS-CoV-2 pseudovirus that is able to simulate the process of the virus entering the cells and then infecting cells, Zhan et al. [55] evaluated the interaction of quercetin and isorhamnetin with ACE2 as well as the inhibitory effect of these two compounds on viral entry. Results demonstrated that, unlike quercetin, isorhamnetin exhibited inhibition on the entrance of SARS-CoV-2 spike pseudotyped virus into ACEh cells in in vitro cell experiments at concentrations that are not toxic to host cells, thus preventing infection of human cells expression ACE2. Khan et al. [56] used both in silico and in vitro approaches to confirm the activity of kaempferol in potentially interacting with the SARS-CoV-2 main protease 3CL^pro^. The docking predictions confirm that kaempferol potentially interacts with the same active site residue even in different conformations, thus verifying its activity against the 3CL^pro^ and in vitro experiment confirms that kaempferol has strong inhibitory effects on 3CL^pro^ (IC_50_ value of 34.46 µg/mL). In a study on the anti-SARS-CoV-2 activity of *Scutellaria baicalensis* and its ingredients, it was reported that both the ethanol extract of *S. baicalensis* and the most active ingredient, baicalein, effectively inhibit the replication of SARS-CoV-2 in cell assay. Liu et al. [67] investigated the structure and activity relationship of baicalein analogues and identified four new active compounds from other herbs that inhibit SARS-CoV-2 3CL^pro^ activity at µM concentration, including myricetin (IC_50_ values of 2.86 µM). Myricetin was also identified as a potent inhibitor of SARS-CoV-2 M^pro^ in another study by molecular docking and enzymatic assay (IC_50_ value of 3.684 ± 0.076 µM). In the same study was demonstrated that myricetin also exhibits a potent anti-inflammation effect on bleomycin-treated mice [66]. Biophysical techniques, such as spectroscopy and calorimetry, were employed to characterize some structural and functional features of SARS-CoV-2 3CL^pro^, and a fast in vitro screening procedure based on 3CL^pro^ hydrolytic activity using a FRET substrate was used by Abian et al. [74] to identify small molecules blocking the enzymatic activity of 3CL^pro^. The natural product quercetin was identified as a reasonably potent inhibitor of SARS-CoV-2 3CL^pro^ (Ki~7 μM).

Liu et al. [75] investigated if flavonoids and other polyphenols with B-ring 3′,4′-hydroxylation inhibit recombinant human (rh)ACE2 activity and found that quercetin, quercetin-3*-O-*galactoside, rutin, quercetin-3*-O-*glucuronide, and polyphenols with 3′,4′-hydroxylation inhibited rhACE2 activity at physiologically relevant concentrations in vitro, with quercetin being the most potent rhACE2 inhibitor among the polyphenols tested, with an IC_50_ of 4.48 μM. It has been demonstrated that both EGCG and theaflavin, the main active ingredients of green tea and black tea, respectively, inhibit 3CL-protease activity in a dose-dependent manner, and the IC_50_ was 7.58 μg/mL for EGCG and 8.44 μg/mL for theaflavin [85]. Furthermore, Henss et al. [86] revealed that EGCG inhibited SARS-CoV-2 virus replication and interfered with the interaction between the SARS-CoV-2 spike protein and ACE2. In silico and in vitro studies demonstrated that hesperidin and its aglycone metabolite hesperetin strongly bind to two proteins that are critical for the cellular entry of SARS-CoV-2: transmembrane serine protease 2 (TMPRSS2) and ACE2. The IC_50_ values of hesperetin and hesperidin in VeroE6 cells were 1491 and 1435 µM, respectively [93]. Naringenin was observed to protect Vero E6 cells from SARS-CoV-2-induced cytopathic effect (CPE) and has been suggested to inhibit SARS-CoV-2 M^pro^ and two-pore channel 2 (TPC2) [96,97].

Abdallah et al. [100] tested in vitro thirty-three plants belonging to seventeen different families used traditionally in Saudi Arabia for their ability to inhibit the SARS-CoV-2 M^pro^. Major constituents of the bio-active extracts were isolated and tested for their inhibitory capacity versus this enzyme as well as against the SARS-CoV-2 Egyptian strain. Among the isolated compounds, only four inhibited the SARS-CoV-2 M^pro^ and, in particular, 6-gingerol showed an inhibition percentage of 65.2%. Moreover, 6-gingerol showed moderate activity against the SARS-CoV-2 virus at non-cytotoxic concentrations in vitro with a significant selectivity index (CC_50_/IC_50_ = 101.3/43.45 = 2.3). A possible mechanism of action for the anti-SARS-CoV-2 activity of curcumin suggested by Goc et al. [103] could be related to the inhibition of binding between SARS-CoV-2 spike (S) glycoprotein and its receptor ACE2. Moreover, curcumin treatment decreased TMPRSS2 activity by about 40% to 50%. In another study, Goc et al. [104] observed that another mode of activity of curcumin could rely on the inhibition of the RdRp viral complex of both SARS-CoV-2 and the Omicron variant. Resveratrol and its analog pterostilbene inhibited the SARS-CoV-2 viral replication up to 48 h post-infection in an in vitro study on air–liquid interface cultured human primary bronchial epithelial cells [108]. Yang et al. [110] reported the effect of resveratrol in replication inhibition in Vero cells previously infected with SARS-CoV-2. Viral replication was measured 48 h after infection, showing a marked inhibition of SARS-CoV-2 replication with an EC_50_ of 4.48 µM. Pasquerau et al. [109] also tested the in vitro effect of resveratrol in SARS-CoV-2 replication and in another coronavirus family member (HCoV)-229E and found that resveratrol significantly decreases the viral replication under non-cytotoxic doses up to 25 µM. A new in vitro RBD-ACE2 binding assay was developed and used to screen two drug libraries for inhibitors that interfere with the RBD-ACE2 interaction. Three compounds that inhibited over 60% of the interaction were selected, including ellagic acid, and were further characterized for their ability to inhibit the infection of Vero E6 cells by a SARS-CoV-2 surrogate virus (rVSV-SARS-CoV-2-S) expressing the SARS-CoV-2 spike protein. No effective inhibition of the rVSV-SARS-CoV-2-S virus could be demonstrated in Vero E6 cells by ellagic acid [111].

It has been demonstrated that sulforaphane exhibits in vitro and in vivo antiviral activity against six strains of SARS-CoV-2, including Delta and Omicron, and seasonal HCoV-OC43 coronaviruses at pharmacologically and potentially therapeutically achievable concentrations [125]. A recent study observed that eugenol interlinked with a spike (S1) protein of SARS-CoV-2 and strongly suppressed the entry of pseudo-type SARS-CoV-2 into human ACE2-expressing HEK293 cells [131]. Moreover, Rizzuti et al. [130] reported that eugenol inhibits the enzymatic activity of 3CL^pro^ with an inhibition constant in the sub-micromolar range (Ki = 0.81 µM).

## 6. In Vivo Studies and Clinical Trials

Until now, to the best of our knowledge, there have been few in vivo studies to evaluate the antiviral activity of natural products or phytochemicals in SARS-CoV-2-infected animal models, especially for the molecules covered in this review. For example, in the aforementioned study by Ordonez et al. [125], the researchers conducted studies in a mouse model of SARS-CoV-2 infection and found that the pre-treatment with sulforaphane resulted in a statistically significant decrease in both the viral load or amount of virus, in the lungs and upper respiratory tract as well as the amount of lung injury compared with infected mice that were not given sulforaphane. Moreover, sulforaphane also decreases inflammation in the lungs, protecting the cells from a hyperactive immune response. Furthermore, in the study of Paidi et al. [131], it has been demonstrated that oral treatment with eugenol reduced lung inflammation, decreased fever, improved heart function, decreased serum markers, and enhanced locomotor activities in SARS-CoV-2 spike S1-intoxicated mice. Anyway, further and more specific experimental and preclinical studies on the effects of these compounds in SARS-CoV-2-infected animal models have to be performed.

Numerous studies have been performed since the beginning of the pandemic to introduce plant extracts and phytochemicals effective in the management of SARS-CoV-2, also in combination with traditional medicines. As an example, in an empirical study conducted at a Wuhan hospital, patients were treated with traditional Chinese medicine remedies, including herbs with a high quercetin content, in addition to conventional therapies. The clinical results showed that patients with coronavirus pneumonia enhanced their immune ability against SARS-CoV-2 with a decrease in hospitalization days [148]. One of the main problems with the use of natural compounds in the treatment of diseases is their low solubility and bioavailability, which makes clinical studies difficult and problematic. Among the ways used to improve drug delivery, biodistribution, biodegradability, and bioavailability of plant-based secondary metabolites, encapsulation or conjugation of these compounds with nanocarriers can be a useful solution. Generally, the most used nanocarriers are organic-based and are basically composed of lipids such as micelles, liposomes, niosomes, bilosomes, solid lipid nanoparticles, and archaeosomes, and these lipid-based drug delivery systems are used to deliver hydrophobic drugs in the body [149].

There are numerous clinical trials investigating the effects of phytochemicals in prophylaxis and the treatment of COVID-19 in which phytochemicals are assessed as pure compounds, pure compounds in combination with other natural bioactive compounds and/or drugs or polyphenol-rich extracts. A list of ongoing or completed clinical trials to evaluate the efficacy of some of the compounds covered by this review in the prevention and as a possible therapeutic management against COVID-19 and registered at ClinicalTrials.gov (accessed on 27 February 2023) is given in Supplementary Table S1.

For example, Di Pierro et al. carried out two different clinical trials to demonstrate the effectiveness of quercetin, especially in the early stages of COVID-19 infection. In particular, in the first trial, supplementation with two doses of 200 mg quercetin daily for 30 days administered to 76 outpatients reduced the number of patients hospitalized (9.2 vs. 28.9%), the days of hospitalization (1.6 vs. 6.8 days), the need for oxygen therapy (1.3 vs. 19.7%) and the aggravation of symptoms compared to the control group [150]. In the second trial, 42 COVID-19 symptom outpatients were divided into two groups, one group receiving standard care treatment while the other group receiving quercetin as a supplemental treatment. Quercetin supplementation not only shortened the timing of molecular test conversion from positive to negative but also reduced the severity of symptoms of COVID-19 [151]. Similarly, in another trial, 120 outpatients received two doses of 250 mg quercetin daily for three months and, also in this case, the number of patients hospitalized (1.67 vs. 6.67%) was lower than in the control group [152]. In a randomized double-blind placebo-controlled proof-of-concept trial of resveratrol for outpatient treatment of mild COVID-19, McCreary et al. administered placebo or resveratrol to 105 participants and found that resveratrol recipients had a lower incidence compared to placebo of hospitalization, COVID-19 related Accident and Emergency (A&E) visits and pneumonia [153].

Many clinical trials have been conducted to evaluate the efficacy of phytochemicals in the prevention and/or treatment of COVID-19 and registered at ClinicalTrials.gov whose results, however, to the best of our knowledge, have not yet been published. For example, a clinical trial has been conducted on 524 volunteer healthcare worker participants with the purpose of determining the efficacy of Previfenon^®^ (epigallocatechin-3-gallate-EGCG) in preventing COVID-19, enhancing systemic immunity, and reducing the frequency and intensity of specific symptoms when used as pre-exposure chemoprophylaxis to SARS-CoV-2. To the best of our knowledge, the stage of this study is still phase 3 [Clinicaltrials.gov I.D. NCT04446065; accessed on 27 February 2023].

A double-blind, placebo-controlled study was carried out in Iran on 40 COVID-19 patients with the aim to identify the effects of nano-curcumin, i.e., curcumin formulated with the aid of nanotechnology in nano-micelles to improve its stability and solubility, on the modulation of inflammatory cytokines, the secretion of which increases significantly in COVID-19 patients. Results demonstrated that nano-curcumin was able to modulate the increased rate of inflammatory cytokines, especially IL-1β and IL-6 mRNA expression [154]. The efficacy of nano-curcumin in the management of 21 mild-to-moderate hospitalized COVID-19 patients was also tested in an open, non-randomized clinical trial. Results demonstrated that most of the symptoms, including fever and chills, tachypnoea, myalgia, and cough, resolved significantly faster in the curcumin group than in the control ones. Moreover, the duration of supplemental O_2_ use and hospitalization was also meaningfully shorter in the treatment group [155]. Nano-curcumin formulation was also administered to mild to moderate COVID-19 patients in the outpatient setting in a randomized triple-blind, placebo-controlled clinical trial to assess the efficacy in the management of the symptoms. The reported results demonstrated that the nanoformulation of curcumin with a dose of 80 mg twice daily could fasten the resolution time of COVID-19-induced symptoms, particularly cough, taste and smell disturbances, chills, and increment of lymphocyte count in comparison with placebo. No substantial adverse reaction was reported in the treatment group [156]. Both of these last two clinical trials have been registered at the Iranian Registry of Clinical Trials with the ID IRCT20200408046990N1. [https://www.irct.ir/trial/47061; accessed on 27 February 2023].

## 7. Conclusions

The rush to publication has not always disseminated results of verified quality, and they should be considered very carefully. For some compounds object of this review, in vitro assays have demonstrated antiviral activity against SARS-CoV-2, also supported by in silico hypotheses on the mechanism of action. Anyway, phenolics consumption helps to modulate the immune system through several mechanisms of action, which significantly influences the prevention of SARS-CoV-2. Clinical trials have demonstrated the effectiveness of SM in the prevention and as a possible therapeutic management against SARS-CoV-2; however, more research is needed. As an example, derivative or chemical modifications of secondary metabolites could be explored as promising compounds.

## Figures and Tables

**Table 1:** List of selected compounds and their anti-viral properties against various viruses and/or SARS-CoV-2.

Compounds	Principal Food Sources	Class	Virus	SARS-CoV-2 Activity
Caffeic acid	Carrots, cabbage, tomatoes, several berries, coffee, basil, thyme, oregano, apples	Phenolic acids	HSV-1, HSV-2, ADV-3 [17,18] HCV [19] IAV [20] HCoV-NL63 [21]	Potential inhibition of SARS-CoV-2 membrane protein M (in silico approach) [22]
Chlorogenic acid	Apples, artichokes, carrots, coffee beans, eggplants, grapes, kiwi fruit, pears, plums, potatoes, tea, tomatoes	Phenolic acids	ADV-3, ADV-8, ADV-11 [18] HCoV-NL63 [21]	Inhibition of SARS-CoV-2 M^pro^ (in silico approach) [23] Inhibition of SARS-CoV-2 replication (in vitro approach) [24]
Ferulic acid	Rice, wheat, oats, pineapple, artichoke, peanuts, and nuts	Phenolic acids		Potential inhibition of SARS-CoV-2 membrane protein M (in silico approach) [22] Anti-inflammatory effects (in vitro approach) [24]
Gallic acid	Blueberry, blackberry, strawberry, plums, grapes, mango, cashew nut, hazelnut, walnut, tea, wine	Phenolic acids	HCV [25] HIV [26] EV71 [27] IAV, IBV [28] HCoV-NL63 [21] HSV [29]	Inhibition of SARS-CoV-2 M^pro^ (in silico approach) [30] Inhibition of SARS-CoV-2 replication (in vitro approach) [24]
p-Coumaric acid	Eggplant, broccoli, asparagus, sweet cherries, plums, blueberries, cranberries, citrus, orange juice	Phenolic acids	HRV-3 [31]	Inhibition of (M^pro^) and RdRp enzymes (in silico approach) [32]
Sinapic acid	Oranges, grapefruits, cranberries	Phenolic acid		Inhibition of SARS-CoV-2 envelope protein E (in silico approach) and inhibition of SARS-CoV-2 replication (in vitro approach) [33]
Hydroxytyrosol	Olives, virgin olive oil, wine	Phenolic alcohols	HIV-1 [34] H1N1, H3N2, H5N1, H9N2, NDV [35]	Interaction with SARS-CoV-2 spike protein and human ACE-2 receptor (in silico approach) [36] Reduction of PL^pro^-dependent adverse effects in long-COVID (in vitro approach) [37]
Apigenin	Parsley, celery, onions, oranges, tea, chamomile, spinach, basil	Flavones	ASFV, HCV, PEDV, FMD virus, HIV, IV, EBV, SARS-CoV (experimental and in silico approach) [38] HBV [39]	Interaction with SARS-CoV-2 NSP10 (in silico approach) [40] Inhibition of SARS-CoV-2 M^pro^ (in silico approach) [41] Inhibition of SARS-CoV-2 M^pro^ (in vitro approach) [42]
Luteolin	Capsicum, carrots, apple, cabbage, onion leaves, parsley, basil, spinach	Flavones	HSV-2 [43] HIV-1, EBV [39] IAV, JEV, DENV, HBV, SARS-CoV [38]	Inhibition of COVID-19 M^pro^ protease (in silico approach) [44,45] Interaction with SARS-CoV-2 RdRp (in vitro approach) [46]
Pectolinarin	Cirsium setidens Nakai	Flavones	SARS-CoV (experimental and in silico approach) [47]	Inhibition of SARS-CoV-2 M^pro^ and spike (S) glycoprotein (in silico approach) [48] Inhibition of SARS-CoV-2 M^pro^ (in silico and in vitro approach) [49]
Rhoifolin	Quinoas, triticales, German camomiles, rice, and oriental wheats	Flavone Glycosides	SARS-CoV (experimental and in silico approach) [47]	Inhibition of SARS-CoV-2 M^pro^ and spike (S) glycoprotein (in silico approach) [48] Inhibition of SARS-CoV-2 M^pro^ (in silico and in vitro approach) [49]
Cirsimaritin	Oregano, lemon verbena, winter savory, rosemary	Dimethoxyflavone	H1N1 [50]	Potential inhibitor against SARS-CoV-2 M^pro^ and ACE2 (in silico approach) [51]
Cyanidin-3-*O*-glucoside	Leafy vegetables, berries, red cabbages, teas, colored grains, plums, black grape	Anthocyanins		Potential inhibitor of SARS-CoV-2 PL^pro^ deubiquitinase activity (in silico and in vitro approach) [52]
Isorhamnetin	Parsley, green bell peppers, dill, romaine lettuces, pears, lemons, chickpeas, apples	Flavonols	A/PR/8/34 (H1N1) [53] EV71, HHV-1, HHV-2, ZIKV * (* in silico approach) [38]	Inhibition of SARS-CoV-2 M^pro^ (in silico approach) [54] Binds to ACE2 receptor (in vitro approach) [55]
Herbacetin	Ephedra sinica Stapf	Flavonols	SARS-CoV (experimental and in silico approach) [47]	Inhibition of SARS-CoV-2 M^pro^ (in silico and in vitro approach) [49]
Kaempferol	Apples, tomatoes, green tea, potatoes, onions, brussels sprouts, lettuce, green and black beans, peaches, blackberries, raspberries, spinach, grapes, broccoli, capers, chives	Flavonols	HCMV, HSV-1, HSV-2, IAV [39]	Potential inhibition of COVID-19 M^pro^ and RdRp enzymes (in silico approach) [32] Inhibition of SARS-CoV-2 M^pro^ (in vitro and in silico approach) [56] Inhibition of SARS-CoV-2 M^pro^ (in silico approach) [57,58,59]
Kaempferol-3-*O*-robinobioside	Opuntia	Flavonols	HSV-1, HSV-2 [60]	Potential inhibition of COVID-19 main protease (in silico approach) [61]
Kaempferol-3-*O*-rutinoside	Red wine, tea, apples, black tea	Flavonols	HSV-1, HSV-2 [60]	Potential inhibition of COVID-19 main protease (in silico approach) [61]
Myricetin	Walnuts, carobs, fennels, welsh onions, yellow bell peppers	Flavonols	HIV [62] IAV [63] HSV [29] SARS-CoV [64]	Inhibition of SARS-CoV-2 M^pro^ (in silico and in vitro approach) [65,66] Inhibition of SARS-CoV-2 M^pro^ (in vitro approach) [67]
Myricetin-3-rhamnoside (Myricitrin)	Summer grapes, blackberry, raspberry, hazelnuts, sapodillas	Flavonols	HIV [68]	Inhibition of SARS-CoV-2 M^pro^ (in silico approach) [65,69]
Quercetin	Apples, berries, grapes, citrus fruits, tea, many seeds, nuts, honey, propolis, radish, fennel	Flavonols	HCV (in silico approach) [70] HSV-1 [71] HIV [72] IAV [73]	Inhibition of SARS-CoV-2 M^pro^ and RdRp enzymes (in silico approach) [30,32,65] Perturbation of the binding of hACE2-S complex (in silico approach) [39,51] Interaction with SARS-CoV-2 NSP16 (in silico approach) [41] Inhibition of SARS-CoV-2 M^pro^ (in vitro approach) [42,74] Inhibition of SARS-CoV-2 M^pro^ (in silico approach) [57,58,59] Interaction with SARS-CoV-2 RdRp (in vitro approach) [46] Inhibition of rhACE2 activity (in vitro approach) [75]
Quercetin 3-*O*-glucuronide	Wine, green beans	Flavonols		Inhibition of SARS-CoV-2 M^pro^ (in silico approach) [65] Inhibition of rhACE2 activity (in vitro approach) [75]
Quercetin-3-beta-galactoside	Walnuts, black chokeberries, red raspberries, summer grapes, almonds	Flavonols	SARS-CoV (in silico approach) [76]	Inhibition of SARS-CoV-2 M^pro^ (in silico approach) [65] Inhibition of rhACE2 activity (in vitro approach) [75]
Quercetin-3-*O*-rutinoside (Rutin)	Parsley, common buckwheats, grape wines, Italian sweet red peppers, nectarines, summer grapes, broccoli, rosemaries, orange, lemon	Flavonols	HCV [77] HSV-1, HSV-2 [60]	Inhibition of SARS-CoV-2 envelope protein E (in silico approach) [22] Inhibition of SARS-CoV-2 M^pro^ (in silico approach) [65] Inhibitor of SARS-CoV-2 PL^pro^ deubiquitinase activity (in silico and in vitro approach) [52] Inhibition of rhACE2 activity (in vitro approach) [75] Inhibition of SARS-CoV-2 replication and anti-inflammatory effects (in vitro approach) [24] Inhibition of SARS-CoV-2 M^pro^ (in vitro approach) [49]
Quercetin-3-*O*-rhamnoside (Quercitrin)	Lingonberries, American cranberries, olives, beans, tea, Welsh onions, bilberries, common pea, apricots, spearmints	Flavonols	A/WS/33 (H1N1) [78]	Inhibition of SARS-CoV-2 M^pro^ (in silico approach) [65]
Catechin	Blackcurrants, blackberries, European plums, redcurrants, cocoa powder. rice, pineapples, cloves, lingonberries, Italian sweet red peppers, argan oil	Flavanols		Inhibition of M^pro^ protein and Spike glycoprotein (in silico approach) [57,79,80,81]
Epicatechin	Pears, star fruits, red tea, common buckwheats, apples, Asian pears	Flavanols	HSV-1, HSV-2 [29] HIV1, HIV2, IAV, IBV [38]	Inhibition of M^pro^ protein and Spike glycoprotein (in silico approach) [79,82]
(-)-Epicatechin-3-*O*-gallate (ECG)	Red tea, herbal tea, green tea, peppermints, common grapes, medlars, kiwifruits, blackberry, raspberry, sweet oranges, common wheats, pistachios	Flavanols	HSV-1, HSV-2 [29] HIV1, HIV2, IAV, IBV [38]	Inhibition of COVID-19 M^pro^ (in silico approach) [57,59] Inhibition of SARS-CoV-2 M^pro^ (in silico approach) [80]
(-)-Epigallocatechin (EGC)	Cocoa beans, green tea, black tea, herbal tea, peanuts, pomegranates, beets, pine nuts, common mushrooms, red bell peppers, allia	Flavanols	HSV [29]	Inhibition of SARS-CoV-2 M^pro^ (in silico approach) [80]
Epigallocatechin-3-gallate (EGCG)	Black and green tea, apples, plums	Flavanols	HBV, HSV, EBV, ADV, HIV, HCV, IV, DENV, JEV, TBEV, ZIKV, CHIKV, HTLV-1, EV71, EBOV, PRRSV, VHSV, IHNV, SVCV [83]	Inhibition of S protein of SARS-CoV-2 (in silico approach) [48] Inhibition of SARS-CoV-2 M^pro^ (in silico approach) [80] Interaction with SARS-CoV-2 M^pro^, RdRp, PL^pro^, S RBD, and ACE2 with S RBD (in silico approach) [84] Inhibition of SARS-CoV-2 M^pro^ (in vitro approach) [85] Inhibition of SARS-CoV-2 RBD/ACE2 binding (in vitro approach) [86]
Theaflavin	Black tea, green tea, herbal tea, red tea	Flavanols	H1N1 (in silico approach) [87]	Inhibition of SARS-CoV-2 M^pro^ (in silico approach) [88] Inhibition of SARS-CoV-2 M^pro^ (in vitro approach) [85]
Hesperetin	Citrus fruits, peppermint	Flavanones	CHIKV [89] YFV (experimental and in silico approach) [90] RSV (experimental and in silico approach) [91] SARS-CoV [92]	Inhibition of SARS-CoV-2 spike protein/ACE2 binding and interaction with TMPRSS2 (in silico and in vitro approach) [93]
Naringenin	Sweet oranges, oregano, sorghums, grape wines, clementine, tangerine, saffrons, white lupines, dates, elderberries	Flavanones	HCV (in silico approach) [70] YFV (experimental and in silico approach) [90] ZIKV (experimental and in silico approach) [94] DENV [95] HSV [29] CHIKV [89]	Inhibition of COVID-19 M^pro^ (in silico approach) [57] Inhibition of SARS-CoV-2 M^pro^ (in vitro approach) [96] Inhibition of TPC2 (in vitro approach) [97]
6-Gingerol	Gingers, cloves, star anises, Ceylon cinnamons, pepper, nutmegs	Gingerols	CHIKV [98]	Interaction with COVID-19 main proteins (in silico approach) [99] Inhibition of SARS-CoV-2 M^pro^ and moderate activity against the SARSCoV-2 virus (in vitro approach) [100]
Curcumin	Turmerics, curry powder, saskatoon berries, peanuts, lettuces, green bell peppers	Curcuminoids	HIV, HSV, HCV, HPV, DENV, ZIKV, CHIKV, HBV, IAV, JEV, MNV, RSV, RVFV [101] EBOV (in silico approach) [102]	Inhibition of COVID-19 M^pro^ (in silico approach) [57,81] Inhibition of SARS-CoV-2 RBD/ACE2 binding; Decrease activity of TMPRSS2 (in vitro approach) [103] Inhibition of RdRP viral complex of both SARS-CoV-2 and the Omicron variant (in vitro approach) [104]
Resveratrol (3,5,4′-trihydroxy-trans-stilbene)	Broccoli, yellow wax bean, turnip, grapes, blueberries, raspberries, mulberries	Stilbenes	HSV-1, HSV-2 [105] HIV-1, PVR, MERS-CoV [39] EV71 [106]	Interaction with SARS-CoV-2 spike protein and human ACE-2 receptor (in silico approach) [107] Inhibition of SARS-CoV-2 replication (in vitro approach) [108,109,110]
Ellagic acid	Raspberries, strawberries, cranberries, walnuts, pecans, pomegranates	Hydrolyzable tannins	HIV-1 [26]	Interaction with SARS-CoV-2 M^pro^ and RdRp enzymes (in silico approach) [32,82] Interaction with SARS-CoV-2 NSP9 (in silico approach) [40] Inhibition of SARS-CoV-2 RBD/ACE2 binding (in vitro approach) [111]
Chebulagic acid	Indian gooseberry	Hydrolyzable tannins	EV71 [112] HSV-2 [113]	Inhibition of SARS-CoV-2 M^pro^ (in vitro and in silico approach) [114]
Punicalagin	Pomegranate	Hydrolyzable tannins	EV71 [115] HSV-2 [116] (experimental and in silico approach)	Inhibition of SARS-CoV-2 M^pro^ (in vitro and in silico approach) [114] Interaction with SARS-CoV-2 S glycoprotein and TMPRSS2 (in silico approach) [117]
Oleuropein	Olives, extra-virgin olive oil, some species of the Oleaceae family	Secoiridoids	RSV, HPIV-3 [118]	Inhibition of COVID-19 M^pro^ (in silico approach) [57,58] Interaction with 3CL^pro^, TLR4, and POP (in silico approach) [119]
Coumarin derivatives	Citrus fruits	Cumarins	HIV-1 *, HCV, IV *, EV71, CHIKV, DENV * (* in silico approach) [120]	Inhibition of SARS-CoV-2 3CL^pro^ (in silico approach) [121] Interaction with NSP12 receptor (in silico approach) [122]
Capsaicin	Green and red peppers, hot chili peppers	Alcaloids	LASV [123]	Inhibition of SARS-CoV-2 M^pro^ (in silico approach) [30,82]
Sulforaphane	Brussels sprout, white cabbage, broccoli, cabbage	Isothiocyanates	IV, HCV, HIV [124]	Inhibition of the in vitro and in vivo replication of SARS-CoV-2 [125]
Allicin	Garlic, onions, shallots, Chinese chives, leeks, saskatoon berry, arrowroot, summer savory	Organosulfur compound	REV, HSV- 1, HSV-2, HPIV-3, VV, VSV, HRV-2 [126]	Inhibition of SARS-CoV-2 M^pro^ (in silico approach) [127]
Eugenol	Cloves, allspices, carrots, walnuts, Ceylon cinnamons, shea tree, passion fruits, winged beans, fireweeds, gingers	Allylbenzenes	HSV-1, HSV-2 [128] IAV, EBOV [129]	Binding affinities towards SARS-CoV-2 spike protein, main protease (M^pro^), RdRp, and human ACE-2 proteins (in silico approach) [128] Inhibition of SARS-CoV-2 M^pro^ (in vitro approach) [130] Interaction with SARS-CoV-2 spike protein and human ACE-2 receptor (in vitro approach) [131]

Abbreviations: ADV (Adenovirus); ADV-3 (Adenovirus type 3); ADV-8 (Adenovirus type 8); ADV-11 (Adenovirus type 11); ACE2 (Angiotensin-converting enzyme 2); A/PR/8/34 (H1N1) (Strain A/Puerto Rico/8/34, Influenza A Virus, Subtype H1N1); ASFV (African Swine Fever Virus); A/WS/33 (Strain A/WS/33, A Virus, Subtype H1N1); BDDV (Bovine viral diarrhea virus); BoHV-1 (Bovine herpesvirus 1); CHIKV (Chikungunya virus); DENV (Dengue virus); EBOV (Ebola virus); EBV or HHV-4 (Epstein–Barr virus); EV71 (Human enterovirus 71); FMD virus (Foot-and-mouth disease virus); H1N1 (Influenza A virus subtype); H3N2 (Influenza A virus subtypes); H5N1 (Influenza A virus subtypes); H9N2 (Influenza A virus subtypes); hACE2-S (Protein complex: human angiotensin converting enzyme-2 receptor and severe acute respiratory syndrome coronavirus 2 spike protein complex); HBV (Hepatitis B virus); HCMV or HHV-5 (Human Cytomegalovirus); HCV (Hepatitis C virus); HCoV-NL63 (Human coronavirus NL63); HIV (Human immunodeficiency virus); HIV-1 (Human immunodeficiency virus type I); HPIV-3 (Human parainfluenza virus type 3); HPV (Human papillomavirus); HRV-2 (Human rhinovirus type 2); HRV-3 (Human rhinovirus type 3); HSV (Herpes simplex virus); HSV-1 or HHV-1 (Herpes simplex virus type 1); HSV-2 or HHV-2 (Herpes simplex virus type 2); HTLV-1 (Human lymphotropic virus type 1); IAV (Influenza A virus); IBV (Influenza B virus); IV (Influenza virus); IHNV (Infectious hematopoietic necrosis virus); JEV (Japanese encephalitis virus); LASV (Lassa virus); MERS-CoV (Middle East respiratory syndrome coronavirus); MNV (Murine norovirus); M^pro^/3CL^pro^ (SARS-CoV-2 Main protease); NDV (Newcastle disease virus); NSP9 (SARS-CoV-2 nonstructural protein 9); NSP10 (SARS-CoV-2 nonstructural protein 10); NSP12 (SARS-CoV-2 nonstructural protein 12); NSP16 (SARS-CoV-2 nonstructural protein 16); PEDV (Porcine epidemic diarrhea virus); PL^pro^ (SARS-CoV-2 papain-like proteases); POP (prolyl oligopeptidases); PRRSV (Porcine reproductive and respiratory syndrome virus); PVR (Pseudorabies virus); RABV (Rabies virus); RBD (Receptor-binding domain); RdRp (RNA-dependent RNA polymerase); REV (Reticuloendotheliosis virus); rhACE2 (Recombinant human Angiotensin-converting enzyme 2); RSV (Respiratory syncytial virus); RVFV (Rift Valley fever virus); S glycoprotein or S protein (Spike glycoprotein); SARS-CoV (Severe acute respiratory syndrome-associated coronavirus); SARS-CoV-2 (Severe acute respiratory syndrome-associated coronavirus 2); TBEV (Tick-borne encephalitis virus); TMPRSS2 (Transmembrane serine protease 2); TLR4 (Toll-like receptor 4); TPC2 (Two-pore channel 2); VEE (Venezuelan equine encephalitis); VHSV (Viral hemorrhagic septicemia virus); VSV (Vesicular stomatitis virus); VV (Vaccinia virus); WNV (West Nile virus); YFV (Yellow fever virus); ZIKV (Zika virus).

## Data Availability

Not applicable.

## Supplementary Materials

The following supporting information can be downloaded at: https://www.mdpi.com/article/10.3390/molecules28062470/s1, Table S1: Clinical trials investigating the effects of selected phytochemicals in prophylaxis and/or the treatment of SARS-CoV-2.
