# Supplementary material for: Food Plant Secondary Metabolites Antiviral Activity and Their Possible Roles in SARS-CoV-2 Treatment: An Overview
Source: Molecules. 2023 Mar 8;28(6):2470. doi: 10.3390/molecules28062470 (PMC10058909; doi:10.3390/molecules28062470)
Supplement: Supplementary file 1 [file molecules-28-02470-s001.zip › molecules-2241485-supplementary.pdf]

Supplementary Table S1 Clinical trials investigating the effects of selected phytochemicals in prophylaxis and/or the treatment of SARS-COV-2

Compounds	Official Title	Intervention/ treatment	Types of Phytochemicals Combinations Used	ClinicalTrials.gov Identifier	References
Caffeic acid<br>Chlorogenic acid<br>Gallic acid<br>p-Coumaric acid<br>Apigenin<br>Luteolin<br>Catechin<br>Epicatechin	Randomized Clinical Trial of Açaí Palm Berry Extract as an Intervention in Patients Diagnosed With COVID-19	Dietary Supplement: Açaí palm berry extract - natural product	Polyphenol-rich extracts	NCT04404218	https://clinicaltrials.gov/ct2/show/NCT04404218?term=caffeic+acid&cond=COVID-19&draw=2&rank=1
Ferulic acid	Retrospective Observational Study to Describe the Evolution of SARS-CoV-2 Disease and the Profile of Patients Treated or Not With Imuno TF® and a Combination of Nutraceuticals and Who Have Tested Positive for COVID-19	Dietary Supplement: ImmunoFormulation	Polyphenols in combination with other natural bioactive compounds	NCT04666753	https://clinicaltrials.gov/ct2/show/NCT04666753?term=Ferulic+acid&cond=COVID-19&draw=2&rank=1
Luteolin	Effects of Palmitoylethanolamide Co-ultramicronized With Luteoline (Pea-lut) on Frontal Lobe Functions and GABAergic Transmission in Long Covid Patients. An 8-week Randomized Controlled Trial	Dietary Supplement: palmitoylethanolamide co-ultramicronized with antioxidant flavonoid luteolin (PEA-LUT)	Polyphenols in combination with other natural bioactive compounds	NCT05311852	https://clinicaltrials.gov/ct2/show/NCT05311852?term=Luteolin&cond=COVID-19&draw=2&rank=1
	Olfactory Disfunction After COVID-19: Conventional Therapy Versus Intervention Treatment With Co-ultraPEALut	Combination Product: co-ultraPEALut	Polyphenols in combination with other natural bioactive compounds	NCT04853836	https://clinicaltrials.gov/ct2/show/NCT04853836?term=Luteolin&cond=COVID-19&draw=2&rank=2
Cyanidin-3-O-glucoside	Dose-response Study a Glucoside- and Rutinoside-rich Crude Material in Relieving Side Effects of COVID-19 Vaccines	Dietary Supplement: Mulberry juice	Polyphenol-rich extracts	NCT05387252	https://clinicaltrials.gov/ct2/show/NCT05387252?term=Cyanidin-3-O-glucoside&cond=COVID-19&draw=2&rank=1

Quercetin	A Prospective, Randomized, Open-labelled, Controlled Trial to Study the Adjuvant Benefits of Quercetin Phytosome in Patients With Diagnosis of COVID-19	Drug: Standard COVID-19 care<br>Dietary Supplement: Quercetin Phytosome	Polyphenols in combination with other natural bioactive compounds and drugs	NCT04578158	https://clinicaltrials.gov/ct2/show/NCT04578158?term=Quercetin&cond=COVID-19&draw=2&rank=1
	The Possible Effect of Quercetin on Prophylaxis and Treatment of COVID-19	Dietary Supplement: Quercetin Prophylaxis<br>Dietary Supplement: Quercetin Treatment	Pure polyphenols	NCT04377789	https://clinicaltrials.gov/ct2/show/NCT04377789?term=Quercetin&cond=COVID-19&draw=2&rank=2
	Study to Investigate the Benefits of Dietary Supplement Quercetin for Early Symptoms of COVID-19	Drug: standard of care for COVID-19 as per the hospital guidelines<br>Dietary Supplement: Quercetin Phytosome (QP)	Polyphenols in combination with other natural bioactive compounds and drugs	NCT04861298	https://clinicaltrials.gov/ct2/show/NCT04861298?term=Quercetin&cond=COVID-19&draw=2&rank=3
	The Study of Quadruple Therapy Zinc, Quercetin, Bromelain and Vitamin C on the Clinical Outcomes of Patients Infected With COVID-19	Drug: Quercetin<br>Dietary Supplement: bromelain<br>Drug: Zinc<br>Drug: Vitamin C	Polyphenols in combination with other natural bioactive compounds and drugs	NCT04468139	https://clinicaltrials.gov/ct2/show/NCT04468139?term=Quercetin&cond=COVID-19&draw=2&rank=4
	Treatment Benefits of Flavonoids Quercetin and Curcumin Supplements for Mild Symptoms of COVID-19	Drug: Standard of care<br>Dietary Supplement: Investigational treatment	Polyphenols in combination with drugs	NCT05130671	https://clinicaltrials.gov/ct2/show/NCT05130671?term=Quercetin&cond=COVID-19&draw=2&rank=5
	Randomized, Placebo-controlled Clinical Trial to Evaluate the Efficacy of an Oral Nutritional Supplement Based on Quercetin in the Prevention of Covid-19 Infection for a Duration of 3 Months	Dietary Supplement: Quercetin	Pure polyphenols	NCT05037240	https://clinicaltrials.gov/ct2/show/NCT05037240?term=Quercetin&cond=COVID-19&draw=2&rank=6

	Complementary Therapy of Dietary Supplements Curcumin, Quercetin and Vitamin D3 for Mild to Moderate Symptoms of COVID-19	Dietary Supplement: Complementary therapy<br>Drug: Standard of care	Polyphenols in combination with other natural bioactive compounds	NCT04603690	https://clinicaltrials.gov/ct2/show/NCT04603690?term=Quercetin&cond=COVID-19&draw=2&rank=7
	The Effectiveness of Phytotherapy in the Treatment of SARS-COV2 (COVID-19)	Drug: Quercetin	Pure polyphenols	NCT04851821	https://clinicaltrials.gov/ct2/show/NCT04851821?term=Quercetin&cond=COVID-19&draw=2&rank=9
	Efficacy of Psidii Guava's Extract For Mild And Symptomless Coronavirus Disease-19 (COVID-19)	Drug: Extract Psidii guava<br>Combination Product: Standard therapy for Covid-19 patient	Polyphenol-rich extracts	NCT04810728	https://clinicaltrials.gov/ct2/show/NCT04810728?term=Quercetin&cond=COVID-19&draw=2&rank=10
	Safety and Efficacy of Hydroxychloroquine for the Treatment & Prevention of Coronavirus Disease 2019 (COVID-19) Caused by Severe Acute Respiratory Syndrome Coronavirus 2 (SARS-CoV-2)	Drug: Hydroxychloroquine<br>Dietary Supplement: Vitamins and Minerals<br>Drug: Azithromycin	Polyphenols in combination with other natural bioactive compounds and drugs	NCT04590274	https://clinicaltrials.gov/ct2/show/NCT04590274?term=Quercetin&cond=COVID-19&draw=3&rank=11
	A Comparative Randomized Clinical Study on Covid-19 Positive Hospitalized Patients Supplemented With NASAFYTOL	Dietary Supplement: NASAFYTOL®<br>Dietary Supplement: FULTIUM® -D3 800<br>Drug: Strandard of care treatment	Polyphenols in combination with other natural bioactive compounds and drugs	NCT04844658	https://clinicaltrials.gov/ct2/show/NCT04844658?term=Quercetin&cond=COVID-19&draw=2&rank=14
Epigallocatechin-3-gallate (EGCG)	A Multicenter, Double-blind, Randomized, Placebo-controlled Clinical Trial to Protect Health Workers Against COVID-19 by Using Previfenon® as Chemoprophylaxis During a SARS-CoV-2 Outbreak. The HERD Study	Drug: Previfenon® (EGCG)	Pure polyphenols	NCT04446065	https://clinicaltrials.gov/ct2/show/NCT04446065?term=Epigallocatechin-3-gallate&cond=COVID-19&draw=2&rank=1

Curcumin	The Effect of a Mixture of Micellized Curcumin/Boswellia Serrata/Ascorbic Acid on Health-related Quality of Life in Patients With Post-acute COVID-19 Syndrome	Dietary Supplement: Curcumin/Boswellia Serrata/Ascorbic acid mixture	Polyphenols in combination with other natural bioactive compounds	NCT05150782	https://clinicaltrials.gov/ct2/show/NCT05150782?term=Curcumin&cond=COVID-19&draw=2&rank=1
	Treatment Benefits of Flavonoids Quercetin and Curcumin Supplements for Mild Symptoms of COVID-19	Drug: Standard of care<br>Dietary Supplement: Investigational treatment	Polyphenols in combination with drugs	NCT05130671	https://clinicaltrials.gov/ct2/show/NCT05130671?term=Curcumin&cond=COVID-19&draw=2&rank=2
	Complementary Therapy of Dietary Supplements Curcumin, Quercetin and Vitamin D3 for Mild to Moderate Symptoms of COVID-19	Dietary Supplement: Complementary therapy<br>Drug: Standard of care	Polyphenols in combination with other natural bioactive compounds	NCT04603690	https://clinicaltrials.gov/ct2/show/NCT04603690?term=Curcumin&cond=COVID-19&draw=2&rank=3
	A Phase III, Double-blind , Controlled Clinical Study Designed to Evaluate the Effect of CimetrA in Patients Diagnosed With COVID-19	Drug: CimetrA-1<br>Drug: CimetrA-2	Polyphenols in combination with other natural bioactive compounds	NCT04802382	https://clinicaltrials.gov/ct2/show/NCT04802382?term=Curcumin&cond=COVID-19&draw=2&rank=4
	A Phase II, Controlled Clinical Study Designed to Evaluate the Effect of ArtemiC in Patients Diagnosed With COVID-19	Drug: ArtemiC	Polyphenols in combination with other natural bioactive compounds	NCT04382040	https://clinicaltrials.gov/ct2/show/NCT04382040?term=Curcumin&cond=COVID-19&draw=1&rank=5
	A Phase IIb, Double Blind, Placebo-controlled Clinical Study Designed to Evaluate the Effect of CimetrA in Patients Diagnosed With COVID-19	Drug: Treatment administration (twice a day)	Polyphenols in combination with other natural bioactive compounds	NCT05037162	https://clinicaltrials.gov/ct2/show/NCT05037162?term=Curcumin&cond=COVID-19&draw=2&rank=6
	Oral Nutritional Supplements in Treatment of Elderly Mild-to-Moderate COVID-19 (ONSITEMC)	Dietary Supplement: Oral Nutritional Supplements	Polyphenols in combination with other natural bioactive compounds	NCT05629975	https://clinicaltrials.gov/ct2/show/NCT05629975?term=Curcumin&cond=COVID-19&draw=2&rank=7

	A Comparative Randomized Clinical Study on Covid-19 Positive Hospitalized Patients Supplemented With NASAFYTOL	Dietary Supplement: NASAFYTOL® Dietary Supplement: FULTIUM® -D3 800 Drug: Strandard of care treatment	Polyphenols in combination with other natural bioactive compounds and drugs	NCT04844658	https://clinicaltrials.gov/ct2/show/NCT04844658?term=Curcumin&cond=COVID-19&draw=2&rank=8
Resveratrol (3,5,4'-trihydroxy-trans-stilbene)	Randomized Double-Blind Placebo-Controlled Proof-of-Concept Trial of Resveratrol, a Plant Polyphenol, for the Outpatient Treatment of Mild Coronavirus Disease (COVID-19)	Drug: Resveratrol Dietary Supplement: Vitamin D3	Polyphenols in combination with other natural bioactive compounds	NCT04400890	https://clinicaltrials.gov/ct2/show/NCT04400890?term=Resveratrol&cond=COVID-19&draw=2&rank=1
	A Pilot Randomized Controlled Clinical Study of Resveratrol for Discharged COVID 19 Patients in Order to Evaluate Its Therapeutic Effects Against Fibrosis	Drug: Resveratrol	Pure polyphenols	NCT04799743	https://clinicaltrials.gov/ct2/show/NCT04799743?term=Resveratrol&cond=COVID-19&draw=2&rank=2
	Can SARS-CoV-2 Viral Shedding in COVID-19 Disease be Reduced by Resveratrol-assisted Zinc Ingestion, a Direct Inhibitor of SARS-CoV-2-RNA Polymerase? A Single Blinded Phase II Protocol (Reszinate Trial)	Dietary Supplement: Zinc Picolinate Dietary Supplement: Resveratrol	Polyphenols in combination with other compounds	NCT04542993	https://clinicaltrials.gov/ct2/show/NCT04542993?term=Resveratrol&cond=COVID-19&draw=2&rank=3
	Retrospective Observational Study to Describe the Evolution of SARS-CoV-2 Disease and the Profile of Patients Treated or Not With Imuno TF® and a Combination of Nutraceuticals	Dietary Supplement: ImmunoFormulation	Polyphenols in combination with other natural bioactive compounds	NCT04666753	https://clinicaltrials.gov/ct2/show/NCT04666753?term=Resveratrol&cond=COVID-19&draw=2&rank=4

	and Who Have Tested Positive for COVID-19				
Oleuropein	Assessment of the Clinical Effectiveness of Standardized Olive Leaf Capsules; as a Co-therapy in the Treatment of Non-hospitalized COVID-19 Patients; a Randomized Clinical Trial	Dietary Supplement: Nusapure standardized olive leaves capsule, 750 mg (50% oleuropein)	Polyphenol-rich extracts	NCT04873349	https://clinicaltrials.gov/ct2/show/NCT04873349?term=Oleuropein&cond=COVID-19&draw=2&rank=1
	Evaluation of the Immunomodulatory and Preventive Effects of Olive Leaf Tea Against COVID-19	Dietary Supplement: Olive Leaf Tea	Polyphenol-rich extracts	NCT05222347	https://clinicaltrials.gov/ct2/show/NCT05222347?term=Oleuropein&cond=COVID-19&draw=2&rank=2
Caffeine	A Non-Randomized, Multiple-Dose, Open-Label, Single Sequence Study To Evaluate The Effect Of Concomitant Administration Of Edp-235 On The Pharmacokinetics And Safety Of Midazolam, Caffeine, And Rosuvastatin In Healthy Participants	Drug: EDP-235<br>Drug: Midazolam<br>Drug: Rosuvastatin<br>Drug: Caffeine	Natural bioactive compounds in combination with drugs	NCT05594615	https://clinicaltrials.gov/ct2/show/NCT05594615?term=Caffeine&cond=COVID-19&draw=2&rank=1
	1,3,7-Trimethylxanthine as a Treatment of COVID-19: Results of a Controlled Study	Other: Data collection	Natural bioactive compounds	NCT04395742	https://clinicaltrials.gov/ct2/show/NCT04395742?term=Caffeine&cond=COVID-19&draw=2&rank=2
Eugenol	Physical Activity and Sensory Trainings to Help COVID-19 Patients Recover From Persistent Smell and Taste Impairments - A Pilot Study	Behavioral: Physical activity<br>Other: Smell training	Natural bioactive compounds	NCT05037110	https://clinicaltrials.gov/ct2/show/NCT05037110?term=Eugenol&cond=COVID-19&draw=2&rank=1
	L'entraînement Olfactif Comme Traitement de la Dysfonction Olfactive Post COVID-19	Other: Smell training	Natural bioactive compounds	NCT05384561	https://clinicaltrials.gov/ct2/show/NCT05384561?term=Eugenol&cond=COVID-19&draw=2&rank=2
	Olfactory Retraining Therapy and Budesonide Nasal Rinse for Anosmia Treatment in Patients	Other: Olfactory retraining	Natural bioactive compounds in combination with drugs	NCT04374474	https://clinicaltrials.gov/ct2/show/NCT04374474?term=Eugenol&cond=COVID-19&draw=2&rank=3

	Post-CoVID 19. A Randomized Controlled Trial	Drug: corticosteroid nasal irrigation<br>Other: smell household Items<br>Other: Nasal Irrigation
